# Harnessing gradient gelatin nanocomposite hydrogels: a progressive approach to tackling antibacterial biofilms†

**DOI:** 10.1039/d3ra06034a

**Published:** 2023-10-17

**Authors:** Jiawei Zhu, Anli Wang, Xingguo Miao, Hui Ye, Shuo Pan, Chengxi Zhang, Qiuping Qian, Feifei Su

**Affiliations:** a Infectious Disease Department, Wenzhou Central Hospital Wenzhou 325099 Zhejiang People's Republic of China feifeisuzs@163.com; b Zhejiang Engineering Research Center for Tissue Repair Materials, Wenzhou Institute, University of Chinese Academy of Sciences Wenzhou 325000 Zhejiang People's Republic of China qianqp@ucas.ac.cn; c Departamento de Química Física, Biomedical Research Center (CINBIO), Universidade de Vigo 36310 Vigo Spain; d Wenzhou Medical University Wenzhou 325000 Zhejiang People's Republic of China; e School of Materials Science and Engineering, Shandong Jianzhu University Jinan 250101 China

## Abstract

Infectious wounds pose significant challenges due to their susceptibility to bacterial infections, hindering tissue repair. This study introduces gradient gelatin nanocomposite hydrogels for wound healing and antibacterial biofilm management. These hydrogels, synthesized *via* UV light polymerization, incorporate copper-doped polydopamine nanoparticles (PDA–Cu) and GelMA (gelatin methacrylate). The hydrogels have a unique structure with a porous upper layer and a denser lower layer, ensuring superior swelling (over than 600%) and effective contact with bacterial biofilms. *In vitro* experiments demonstrate their remarkable antibacterial properties, inhibiting *S. aureus* and *E. coli* biofilms by over 45% and 53%, respectively. This antibacterial action is attributed to the regulation of reactive oxygen species (ROS) production, an alternative mechanism to bacterial cell wall disruption. Moreover, the hydrogels exhibit high biocompatibility with mammalian cells, making them suitable for medical applications. *In vivo* evaluation in a rat wound infection model shows that the gradient hydrogel treatment effectively controls bacterial biofilm infections and accelerates wound healing. The treated wounds have smaller infected areas and reduced bacterial colony counts. Histological analysis reveals reduced inflammation and enhanced granulation tissue formation in treated wounds, highlighting the therapeutic potential of these gradient nanocomposite hydrogels. In summary, gradient gelatin nanocomposite hydrogels offer promising multifunctional capabilities for wound healing and biofilm-related infections, paving the way for innovative medical dressings with enhanced antibacterial properties and biocompatibility.

## Abstract 2

Gradient gelatin nanocomposite hydrogels based on PDA–Cu nanoparticles and PgelMA hydrogels with high biocompatibility with mammalian cells, making them suitable for wound healing and antibacterial biofilm management.

## Graphical abstract

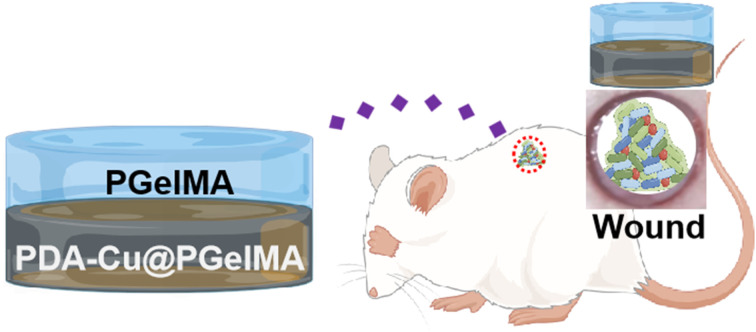

## 1. Introduction

Chronic wounds caused by bacterial infections have become a major challenge in the field of medical care, especially skin wounds infected with bacteria such as *Staphylococcus aureus*.^1–5^ These infections often lead to significant tissue damage and hinder the healing process. Therefore, there is an urgent need to develop wound dressings with antibacterial properties targeting bacterial biofilms.

Currently, antibiotics are widely used in clinical treatments to control infections.^6^ However, this practice frequently results in the development of antibiotic-resistant bacteria, complicating subsequent treatments and potentially harming patients even more.^6,7^ As a result, finding more effective methods to prevent or eliminate bacterial biofilm wound infections has become a critical issue. Copper ions have gained attention due to their excellent physicochemical properties and broad-spectrum antibacterial capabilities.^8–11^ They have emerged as a promising material for developing antibacterial alternatives and have been widely incorporated into wound dressings.^12,13^ However, copper ions are prone to depletion, with their antibacterial activity lasting for a limited time. Additionally, the high concentration of copper ions may lead to toxic side effects.^14^ Therefore, there is a pressing need for wound dressings capable of sustained release of copper ions. Nano materials, owing to their tunable size and controllable morphology, offer a favorable platform for the loading of metal ions.^15–17^ Polydopamine (PDA), an important natural melanin-like substance, has garnered attention due to its ease of fabrication, exceptional biodegradability, superior biocompatibility, and photostability.^18,19^ Utilizing PDA for effective copper ion loading represents a technological breakthrough and holds promise for constructing novel wound dressings.^20,21^

In order to facilitate the healing of infectious wounds, various biomaterials have been developed, including electrospun fibers, foams and sponges, films, and hydrogels.^22–26^ Among these, hydrogels hold great promise in wound dressing applications due to their three-dimensional network structure, wound exudate absorption capacity, moisturizing ability, and oxygen permeability.^27–29^ However, existing hydrogel dressings struggle to meet the demands of preventing bacterial biofilms, necessitating the design of hydrogel wound dressings with both excellent biocompatibility and antibacterial biofilm properties.

Although collagen, derived from partial hydrolysis of the natural polymer collagen protein, possesses numerous advantageous properties such as high biocompatibility, non-irritation to the skin, high absorption rates of tissue exudates, ideal cell adhesion properties, and moisture retention abilities,^30,31^ it is limited by its poor mechanical properties and lack of intrinsic antibacterial properties. Gelatin-methacryloyl (GelMA), achieved through methacrylation, has been introduced to confer new functionalities, enhancing mechanical strength.^32^ However, addressing the critical issue of eradicating bacteria embedded in biofilms at wound sites poses a significant challenge for GelMA itself. This challenge is often overlooked in design, yet it remains a central issue in achieving effective wound healing.

Considering this, nanocomposite materials present a promising avenue to address these challenges, combining the favorable properties of nanoparticles and organic matrices.^33–35^ However, achieving good compatibility between inorganic components and organic matrices remains a substantial technical hurdle.

**Fig. 1:**
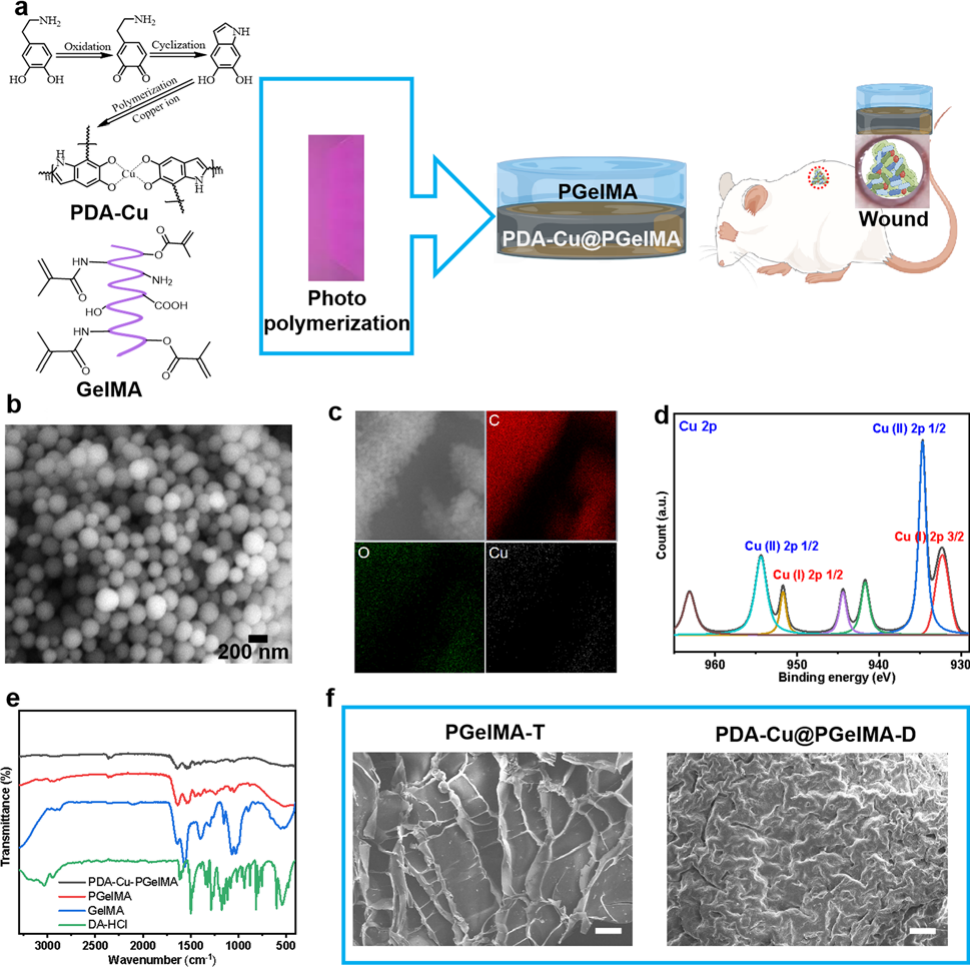
Preparation and characterization of gradient gelatin nanocomposite hydrogels. (a) Schematic illustration for the preparation of nanocomposite hydrogel for infectious wound healing, the purple rectangle used to represent ultraviolet radiation. (b and c) SEM and element mapping images of PDA–Cu nanoparticles. (d) High-resolution XPS spectra of Cu 2p for PDA–Cu@PgelMA. (e) FTIR spectra of unit components of nanocomposite hydrogel. (f) SEM images of gradient gelatin nanocomposite hydrogel, scale bar: 100 μm.

Hence, in this study, we propose a gradient composite hydrogel based on copper-doped nanoparticles and gelatin-methacryloyl (GelMA). By ensuring excellent biocompatibility, this hydrogel not only demonstrates remarkable mechanical properties but also effectively combats bacterial biofilms (Fig. 1). The innovation not only offers a novel approach for treating infectious wounds but also opens up new possibilities for the application of smart hydrogels as wound dressings.

## 2. Experimental section

### 2.1. Materials

All the reagents for the synthesis of PDA nanoparticles doped by Cu ions were purchased from Aladdin Reagent Co. Ltd., China, including dopamine hydrochloride, ammonia, sodium hydroxide, copper chloride dihydrate, ethanol. Low endotoxin GelMA and lithium phenyl-2,4,6-trimethylbenzoylphosphinate (LAP) were obtained from Sigma-Aldrich Co. Ltd., China. Fetal bovine serum (FBS) and Dulbecco's modified Eagle's medium (DMEM) were from Gibco, USA. Calcein/propidium iodide (PI) Cytotoxicity Assay Kit was from Beyotime, China. Luria–Bertani broth (LB), Nutrient Broth (NB) and tryptone soya broth (TSB), were supplied by Solarbio Co., China. Purple crystal, alcohol (≈95%) and PBS buffer were purchased from Aladdin. *Escherichia coli* (*E. coli*, ATCC 8739) and *Staphylococcus aureus* (*S. aureus*, ATCC 12600) were obtained from Shanghai Bioresource Collection Center (SHBCC). Cell Counting Kit-8 was purchased from Dongren Chemical Technology (Shanghai) Co., LTD. Deionized water (18.2 MΩ cm^−1^) was obtained from a Milli-Q water-purification system. The other chemicals were used as purchased without further purification.

### 2.2. Synthesis of PDA–Cu nanoparticles (NPs)

The PDA–Cu NPs were prepared by dissolving 0.5 grams of dopamine hydrochloride (DA·HCl) in a mixture of 50 mL of ethanol and 50 mL of deionized water, containing 0.15 grams of copper chloride dihydrate. The oxidation reaction was initiated by adding 2 mL of concentrated ammonia and carried out under ambient conditions (25 °C) with continuous magnetic stirring for 24 hours. Subsequently, the PDA–Cu NPs were subjected to centrifugation at 9000 rpm for four cycles, each lasting 20 minutes, followed by lyophilization to obtain a fine powder.

### 2.3. Preparation of gradient gelatin nanocomposite hydrogels

GelMA solutions (5% wt and 10% wt) were mixed with phosphate-buffered saline (PBS) solution at 45 °C for 60 minutes, resulting in the formation of solution A and solution B, respectively. Simultaneously, PDA–Cu solutions (1 mg mL^−1^ and 10 mg mL^−1^) were mixed with phosphate-buffered saline (PBS) to obtain solution C and solution D, respectively. Next, LAP (20 mg mL^−1^) was dissolved in PBS/ethanol (1 : 1 wt) to create solution E. For the preparation of the 5% GelMA hydrogel (PgelMA), 1 mL of solution A and 10 μL of solution E were added to a polytetrafluoroethylene (PTFE) mold using a pipette. The homogeneous solution was then cured under UV light (300 W) for 300 seconds to form the hydrogel. The procedure for preparing the 10% PgelMA was similar to the one described above. To create PDA–Cu@PgelMA-01, 0.7 mL of solution B, 0.3 mL of solution C, and 10 μL of solution E were pipetted into a PTFE mold. The resulting homogeneous solution was cured under UV light (300 W) for 300 seconds to produce the hydrogel. PDA–Cu@PgelMA-02 was fabricated similarly, utilizing solution B, D, and E. The gradient gelatin nanocomposite hydrogel PgelMA-T/PDA–Cu@PgelMA-D was prepared by adding 0.35 mL of solution B, 0.15 mL of solution C, and 5 μL of solution E into a PTFE mold using a pipette. The homogeneous solution was cured under UV light (300 W) for 300 seconds, resulting in the formation of the bottom layer hydrogel (PDA–Cu@PgelMA-D). Then, 0.5 mL of solution B and 5 μL of solution E were added to the bottom layer hydrogel, and the homogeneous solution was again cured under UV light (300 W) for 300 seconds to create the top layer hydrogel (PgelMA-T). The preparation procedure for the gradient gelatin nanocomposite hydrogel of PDA–Cu@PDA–Cu@PgelMA-T/PgelMA-D was analogous to the one described above (Table S1†).

### 2.4. Scanning electron microscope

To analyze the microstructure of the biohydrogels, freeze-dried and fractured hydrogel samples were observed using emission scanning electron microscopy (SEM) with a Hitachi Regulus 8100 instrument from Japan, operating at 3 kV. Prior to examination, the samples were coated with a layer of platinum.

### 2.5. FTIR analysis

The hydrogels were evaluated by Fourier Transform Infrared (FTIR) spectroscopy (Nicolet iS 10). The measurements were carried out at room temperature over the wavenumbers ranging from 500 to 4000 cm^−1^.

### 2.6. Rheological studies

The rheological behavior of hydrogels was studied using a rheometer (Thermo Fisher Mars 40) in the oscillatory mode using a parallel plate (diameter of 8 mm) at 25 °C. The frequency sweep tests were carried out in the predetermined linear viscoelastic region (the strain of 0.1%) ranging from 0.01 to 10 Hz. To prevent water evaporation during the measurements, silicon oil was applied at the periphery of the samples.

### 2.7. Hygroscopicity of hydrogels

Following the procedure outlined above, 1 mL hydrogels were synthesized in three portions for each group. Upon freeze-drying, the dry weights (*W*_0_) of the hydrogels were measured, and subsequently, 10 mL of PBS was added to each sample. These wet hydrogels were then incubated at 37 °C and weighed at specific time intervals: 1 hour, 12 hours, 24 hours, and 48 hours (*W*_*t*_). The swelling ratio was determined using the formula: [(*W*_*t*_ − *W*_0_)/*W*_0_] × 100%.

### 2.8. *In vitro* cytotoxicity assay of gels

Cell toxicity of gels was evaluated by CCK-8 assay. Briefly, mouse epithelioid fibroblasts (L929) cell were seeded into a 24-well Transwell plate at ∼10 000 cells per well. The gels were soaked in the medium for different duration, such as 12 hour (*n* = 3), and the 24-wells plates were placed in the incubator for 24 h at 37 °C and 5% CO_2_. Next, the supernatant was removed, and a 10% volume of CCK-8 solution in a serum-free medium was introduced into the cells. The absorption value of each well was measured using a microplate reader (Thermo Scientific Varioskan LUX) after incubation for two hours.

### 2.9. Antibacterial property of PDA–Cu

*Staphylococcus aureus* (10^5^ CFU mL^−1^*S. aureus*) and *Escherichia coli* (10^5^ CFU mL^−1^*E. coli*) were divided into two groups. In the first group, they were incubated with a PDA–Cu PBS solution for 1 hour. The second group served as a blank control. Following this incubation, *S. aureus* was inoculated onto an agar plate at a density of 10^4^ CFU mL^−1^, and *E. coli* was similarly inoculated at a density of 10^4^ CFU mL^−1^. After incubation for either 18 or 24 hours, photographs were taken for subsequent analysis.

### 2.10. Preparation of nutrient broth medium (1× NB)

18 g NB was added into 1000 mL distilled water, after which the solution was boiled and dissolved, and autoclaved at 121 °C for 15 minutes. The preparation of LB, BHI, and TSB medium was similar to operations as above.

### 2.11. Biofilm formation assay

The quantification of the formed biofilm was conducted utilizing a microtiter dish method, involving the development of a biofilm on both the wall and the bottom of the Transwell microtiter dish. To assess the impact of the gels placed in the Transwell microtiter dish on biofilm formation, bacterial suspensions (at a concentration of 10^5^ cells per mL for *E. coli* in 1× NB and 10^5^ cells per mL for *S. aureus* in 1× TSB) were incubated in 24-well plates at 37 °C in the absence of light for 24 hours, allowing for the formation of biofilms, parallel experiments were carried out in four separate groups.

Following the formation of the biofilm, the bacterial nutrient solution was aspirated from the plates. 4 mm × 4 mm gels from the Transwell microtiter dishes were then incubated with the formed biofilms for 60 minutes. Subsequently, the dishes were gently washed twice with water to eliminate unattached bacterial cells and media components. Afterward, the gels were detached from the biofilms. The biofilms were dried overnight in ambient air, and each well received 0.6 mL of acetic acid (30% v/v in water) to dissolve the crystal violet absorbed by the bacterial biofilms. The solubilized crystal violet was assessed at an optical density of 550 nm (OD_550_) using a Thermo Scientific Varioskan LUX Multimode Microplate Reader. For the examination of biofilm morphology, the formed biofilms were stained using a LIVE/DEAD BacLight Bacterial Viability Kit and visualized using an Olympus FV1200 confocal laser scanning microscopy imaging system.

### 2.12. *In vivo S. aureus* biofilm-infected model

All experimental animals were handled according to the guidelines formulated by the National Institutes of Health on human use and care of laboratory animals. All procedures performed on animals were approved by the Animal Care and Use Committee of Wenzhou Institute, University of Chinese Academy of Sciences.

Eight-week-old Sprague-Dawley (SD) rats weighing between 250–300 grams were randomly selected for the wound infection model. To create a *S. aureus* biofilm-infected skin model, rats were anesthetized with pentobarbital sodium. The model was constructed by removing the back hairs and creating a full-thickness wound, approximately 10 mm in diameter, on the upper back of the rats using a round punch. These wounds were then infected with 100 μL of 10^8^ CFU mL^−1^*S. aureus* for 24 hours to establish the infected wound. The rats were divided into three treatment groups: one treated with PBS, another with PDA–Cu@PgelMA-T/PgelMA-D, and the third with PgelMA-T/PDA–Cu@PgelMA-D. Throughout the experimental period, all treated rats were housed separately and closely monitored. Following treatment, wound tissue was dissected and placed in an equal volume of sterile PBS. The number of *S. aureus* was determined by measuring the bacterial load in the infected wound area on day 4. Sterile cotton swabs were employed to swab the wound, and the swabs were then washed with PBS to collect the samples. The bacterial cell burden on each rat was assessed using the LB-agar plate dilution method.

Histopathology assessment: upon completion of the treatment, histological analysis of the *S. aureus*-infected wound tissue was conducted. The rats were euthanized by intraperitoneal injection of pentobarbital sodium (70–100 mg per kg body weight). Subsequently, wound tissue and organs from rats in different groups were fixed in a 4% formalin solution and embedded in paraffin. Tissues were sectioned into 3 μm thick sections. These sections were then dewaxed in xylene and gradually dehydrated using a series of ethanol solutions. Finally, the sections were stained with hematoxylin and eosin (H&E), subjected to immunohistochemistry, and scanned using a Pannoramic NIDI (3D HISTECH) system for further analysis.

### 2.13. Statistical analysis

All the quantitative data were analyzed by the *t*-test, one-way ANOVA with Dunnett's multiple comparison test, or two-way ANOVA with Dunnett's multiple comparison test. The data were presented as mean with s.d. values of *p < 0.05, **p < 0.01, ***p < 0.001 and ****p < 0.0001 were considered statistically significant.

## 3. Results and discussion

### 3.1. Synthesis and characterization of the gradient gelatin nanocomposite hydrogels

The typical process for preparing gradient nanocomposite hydrogels using ultraviolet (UV) light polymerization is as shown in the Fig. 1, which are composed of simple components PDA–Cu and GelMA. These components, along with the UV initiator, are dissolved separately in deionized water and then mixed to form a homogeneous solution at 500 rpm stirring. Subsequently, the homogeneous solution is sequentially added to a polytetrafluoroethylene (PTFE) mold using a pipette. The homogeneous solution is then cured under ultraviolet (UV) light for 600 seconds, resulting in the formation of gradient nanocomposite hydrogels. PDA–Cu and GelMA maintain the hydrogen bonds and physical cross-linking interactions between molecules. This enhances the mechanical strength significantly, enabling the gradient hydrogel to be removed from the mold without any damage. In gradient nanocomposite hydrogels, the size of the nanoscale unit PDA–Cu is 149.80 ± 38.13 nm, primarily composed of carbon, oxygen, nitrogen, and copper elements, as shown in Fig. 1b, c and S1.† The copper content is about 6.19% (Table S2†).

X-ray Photoelectron Spectroscopy (XPS) was further performed to explore the surface composition, and a full scan spectrum confirmed that the PDA–Cu@PgelMA mainly consisted of elements of carbon, oxygen, nitrogen and copper, as shown in Fig. S1.† To further investigate the chemical state of copper (Cu) in the gradient gelatin nanocomposite hydrogel, XPS analysis was conducted. As anticipated, the Cu XPS spectra of the sample was analyzed using Gaussian curve-fitting. Two peaks were observed: one centered at 934.81 eV (2p 3/2) and 954.2 eV (2p 1/2), indicating the presence of Cu(ii) in the PDA–Cu–PgelMA component, while the other peak at 932.21 eV (2p 3/2) and 951.96 eV (2p 1/2) suggested the existence of Cu(i) in the hydrogel. The ratio of monovalent copper to divalent copper was approximately 45.2%. Additionally, the XPS spectra in the Cu 2p region exhibited some strong Cu(ii) shake-up satellites at 941.93 eV, 944.45 eV, and 962.99 eV. Fourier-transform infrared spectroscopy (FT-IR) was conducted to provide further confirmation that PDA–Cu@PgelMA contained the main characteristic peaks of both PDA–Cu and GelMA. This indicated that PDA–Cu was effectively incorporated into the nanocomposite hydrogels. Notably, various characteristic peaks, especially those associated with C=C in GelMA, were no longer observed in the gradient hydrogels. This disappearance of these peaks signified the successful polymerization process, resulting in the formation of a high-quality hydrogel, as shown in Fig. 1e. Scanning electron microscopy (SEM) was employed for morphological analysis of the upper and lower layers of the gradient nanocomposite hydrogel (Fig. 1f). The hydrogel was freeze-dried, and cross-sectional analyses were conducted on both layers. It can be observed that the cross-section of the upper layer of the hydrogel exhibited a uniform and porous three-dimensional network structure ranging from 50 nm to 800 μm, with a calculated porosity of 65.18% for PgelMA (top layer) using ImageJ. In contrast, the lower layer of the hydrogel showed smaller and slightly denser pores, with a calculated porosity of 35.66% for PDA–Cu@PgelMA (down layer) using ImageJ. The porous structure of the hydrogel mimics the extracellular matrix (ECM), which is beneficial for skin cell proliferation and differentiation. Additionally, the presence of antibacterial functional units like PDA–Cu in the lower layer provides protection against bacterial biofilm formation, which is crucial for the successful healing of wounds in the infected skin area using such gel dressings.

### 3.2. Swellability and mechanical properties of the gradient gelatin nanocomposite hydrogels

**Fig. 2:**
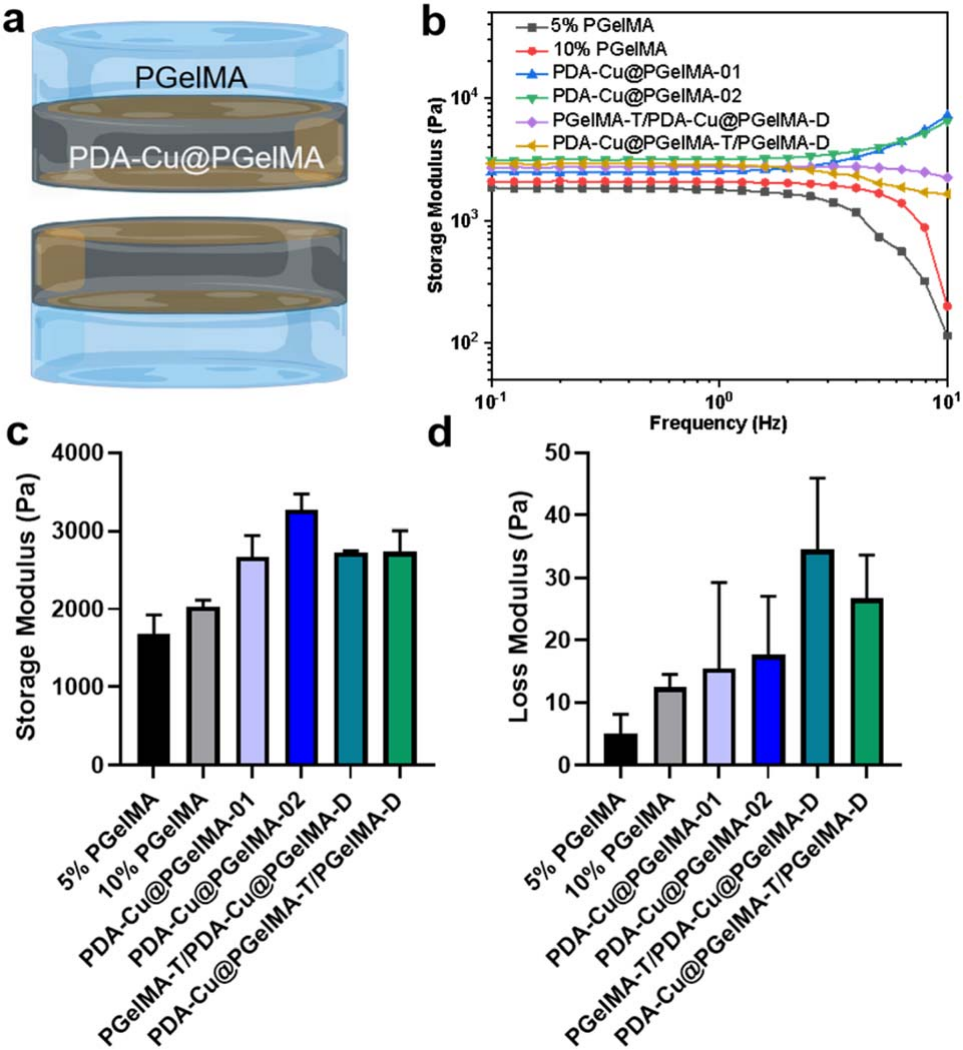
The mechanical properties of the hydrogels: (a) schematic diagram of the structure of gradient gelatin nanocomposite hydrogels. (b and c) The storage modulus of hydrogels. (d) The loss modulus of hydrogels.

In addition to having sufficient space and a good-strength structure, gradient composite biohydrogels can more effectively interact with antibacterial units within the bacterial microenvironment. This is achieved through their swelling characteristics. After incubation in phosphate-buffered saline (PBS), the hydrogel rapidly expands within 24 hours, ultimately reaching a swelling ratio exceeding 600% (Fig. S2†). This not only provides a hydrated environment for tissue cells in the infection area but also ensures the feasibility of the antibacterial membrane. Furthermore, we conducted oscillatory rheological studies on the gradient gel and its individual components to further investigate the mechanical properties of the samples. As shown in Fig. 2, the storage modulus (*G*′) consistently exceeded the loss modulus (*G*′′), confirming the elastic behavior of all hydrogels under applied strain. Additionally, both PDA–Cu@PgelMA-02 and the gradient gel exhibited superior mechanical performance compared to PgelMA and PDA–Cu–PgelMA-01. Statistical analysis results demonstrated that the gradient gel PgelMA-T/PDA–Cu–PgelMA-D or PDA–Cu–PgelMA-T/PgelMA-D, showed significantly better *G*′ and *G*′′ performance among all groups (Fig. 2c and d). PDA–Cu not only functions as a physical cross-linking site but also forms robust hydrogen bonds with GelMA, enhancing the mechanical properties of the nanocomposite gels. This indicates that the gradient composite gel possesses a high-strength structure, effectively serving as a physical barrier to cushion traumatic tissue, thereby offering potential for future biological applications.

### 3.3. *In vitro* antibacterial biofilm activities of the gradient gelatin nanocomposite hydrogels

Biofilm-related infections are commonly considered as the major cause of chronic inflammation and recurrent infections. We conducted additional experiments with two gradient gels and a control group to assess which hydrogel exhibited superior antibacterial biofilm effects, with untreated biofilms serving as the control group.

**Fig. 3:**
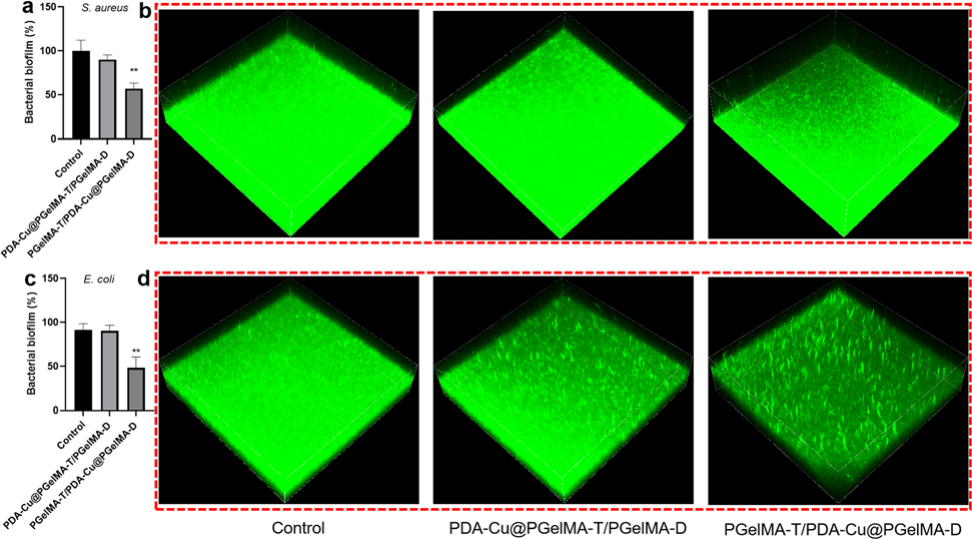
Assessment of antibacterial biofilm activity: quantitative evaluation of *S. aureus* (a) or *E. coli* (b) biofilm inhibition following treatment with gradient gelatin nanocomposite hydrogels by measuring the OD_550_ of crystal violet-treated biofilms. 3D CLSM images depicting biofilm disruption effectiveness in the absence of hydrogels, after PDA–Cu–PgelMA-T/PgelMA-D or PgelMA-T/PDA–Cu–PgelMA-D treatments against *S. aureus* (c) or *E. coli* (d) post-staining with SYT09 (each CLSM image size: 1272 μm × 1272 μm × 150 μm).

**Fig. 4:**
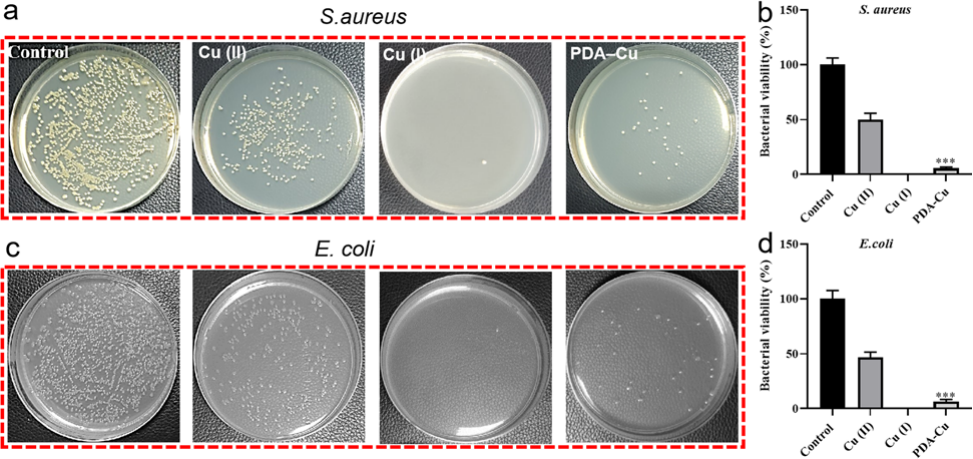
*In vitro* antibacterial activities of PDA–Cu. The PDA–Cu (0.1 mg mL^−1^), Cu(ii) 5 μM or Cu(ii) 5 μM against *S. aureus* (a) and *E. coli* (c) by the agar plate method to evaluate the antibacterial effect. Histograms of antibacterial activity against *S. aureus* (b) and *E. coli* (d), represented by the ratio of different groups to control groups, the error bars indicate standard deviations in experiments with three samples.

We quantified the biofilm mass by recording the OD_550_ of crystal violet-stained biofilms in microtiter plates. Additionally, these biofilms were stained with SYTO9 (LIVE dye) and observed through confocal laser scanning microscopy (CLSM), where deeper green coloration indicated more live bacteria. As shown in Fig. 3a–d, the PgelMA-T/PDA–Cu–PgelMA-D hydrogel demonstrated higher effectiveness in inhibiting both *S. aureus* and *E. coli* biofilms, with inhibition efficiencies of approximately 45% and 53%, respectively. This performance was significantly superior to that of the PDA–Cu–PgelMA-T/PgelMA-D gel treatment. To investigate the mechanism behind the antibacterial biofilm properties of the gradient composite gel, we conducted experiments using Gram-positive bacteria (*S. aureus*) and Gram-negative bacteria (*E. coli*) to assess the effectiveness of the antibacterial unit PDA–Cu and the influence of copper ions in various valence states. Quantitative analysis was performed using colony forming units (CFU) on agar plates to gauge their impact on bacteria. The agar plate colony images and corresponding quantitative histogram data are presented in Fig. 4. After a 1 hour exposure to PDA–Cu, the survival rate of bacterial cells was remarkably low. *S. aureus* and *E. coli* exhibited survival rates of less than 6% and 8%, respectively.

**Fig. 5:**
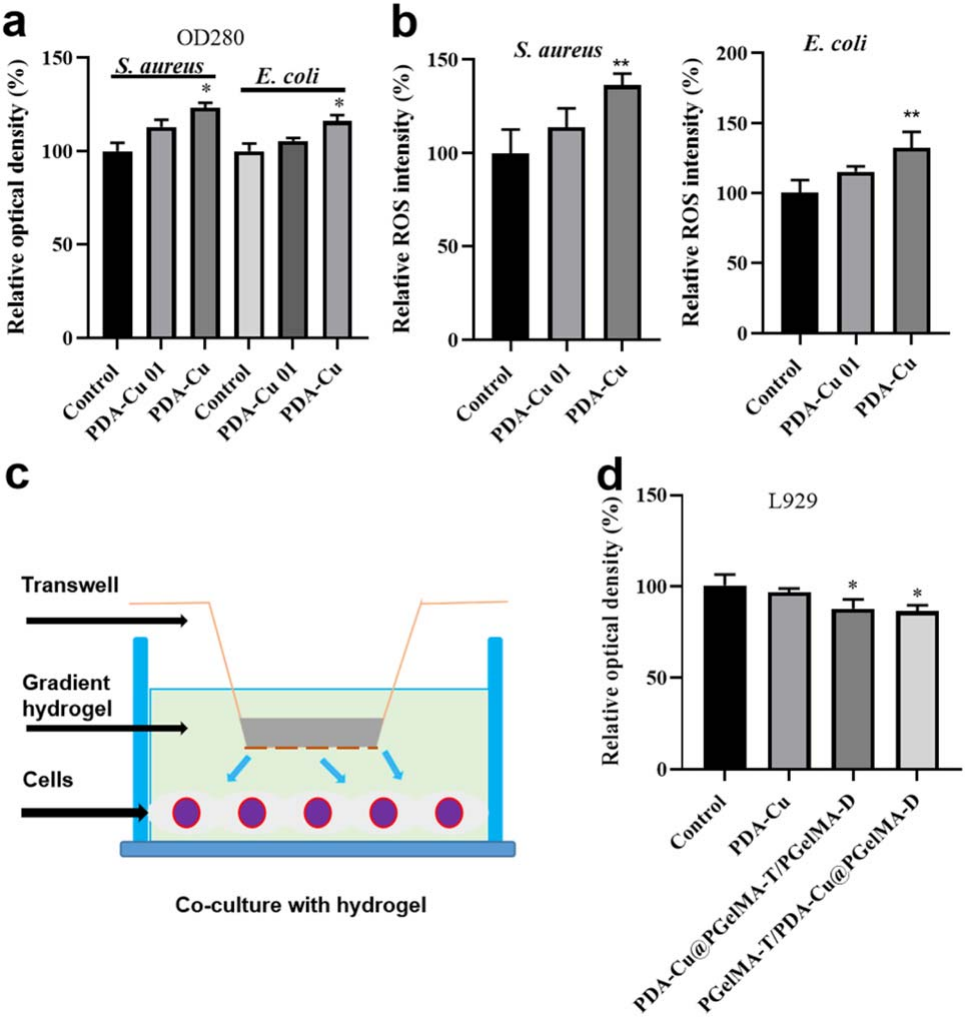
Gradient gel antibacterial mechanism and cytocompatibility. (a) The content of proteins leakage in *S. aureus* or *E. coli* culture medium quantified after treatment with different concentrations of PDA–Cu (0.1 mg mL^−1^, 0.5 mg mL^−1^). (b) Reactive oxygen species in bacterial cells exposed to different concentrations of PDA–Cu were detected *via* reactive oxygen species assay kit. Cellular compatibility assessment of gradient nanocomposite hydrogels, (c) illustrates the concept of cell co-cultivation with hydrogels. (d) CCK-8 assays were performed on L929 cells co-cultured with equal volumes of PBS and hydrogels after one day (*n* = 3).

These antibacterial effects fell between those of divalent and monovalent copper ions. This observation aligns with our previous XPS data analysis. Copper ion sterilization primarily relies on disrupting bacterial cell walls and membranes. When the bacterial structure is compromised, intracellular components such as DNA, RNA, and proteins are released from the cell. We employed a microplate reader to quantify the relative concentration of extracellular proteins in bacteria following PDA–Cu treatment. As the dosage increased, there was a corresponding increase in protein concentration (Fig. 5a).

### 3.4. Antibacterial mechanism and biocompatibility of hydrogels

We found that damage to the bacterial cell walls and membranes is not the sole mechanism for bacterial cell death. PDA–Cu can also regulate the production of ROS to kill bacterial cells. As shown in Fig. 4b, it induces the excessive production of ROS in both *S. aureus* and *E. coli*. As expected, the relative fluorescence intensity and ROS levels in bacterial cells increase with the increasing concentration of PDA–Cu. Additionally, biocompatibility is a crucial requirement for hydrogels. Therefore, we employed CCK-8 assays to assess the cell compatibility of the hydrogel. We conducted further analysis using a Transwell co-culture system and cell counting with CCK-8. After exposure to the gradient gel, cell viability exceeded 85%, indicating that the gradient nanocomposite hydrogel does not exhibit significant toxicity towards normal mammalian cells. We proceeded to implant the PgelMA-T/PDA–Cu–PgelMA-D hydrogel under the skin of rats. After one week, we conducted a cross-sectional analysis of the major organs, including the liver, heart, spleen, lung, and kidney, using hematoxylin and eosin (H&E) staining. The results revealed that the rats were in a normal and healthy condition, indicating that this hydrogel has minimal side effects (Fig. S3†).

### 3.5. Infected wound healing evaluation *in vivo*

**Fig. 6:**
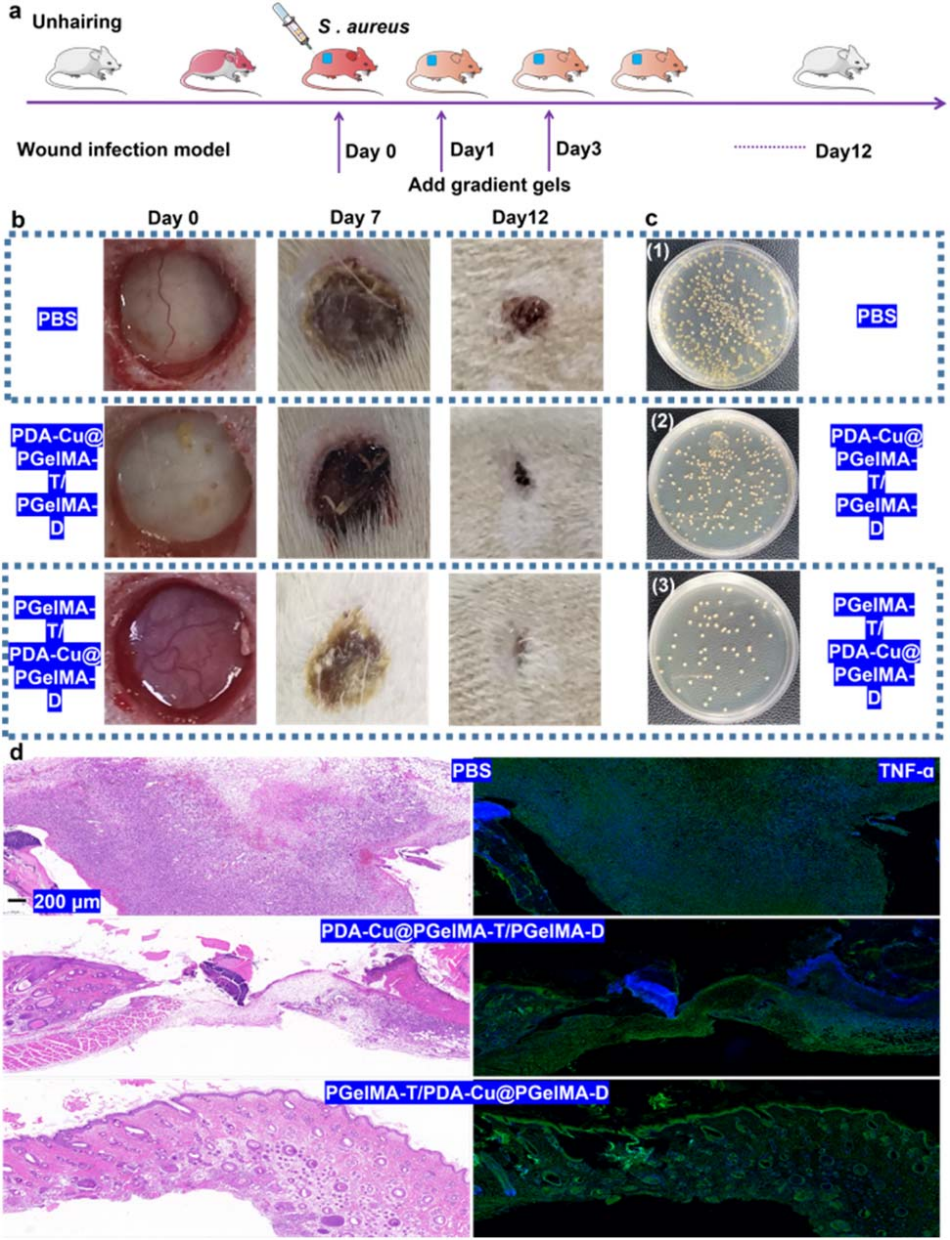
Evaluation of wound infection by bacterial biofilm *in vivo*. (a) Schematic diagram of skin wound infection with *S. aureus* and treatment with gradient nanocomposite hydrogels. Rats were evaluated on the 10th day. (b) Wound area changes in *S. aureus* – infected rats from 0 to 12 days during the treatment. (b) Representative images of wound changes in *S. aureus* – infected rats after treatment with nanocomposite hydrogels within twelve days. (c) Evaluation of bacterial colonies inside the infected skin after 6 d treatment. (d) Microscopy images of hematoxylin and eosin (H&E) stained sections of wound tissues. Infected skin wound changes evaluated tissue in TNF-α (green) during treatment in rats by immunohistochemistry, scale bar: 200 μm.

In order to further assess the potential of gradient nanocomposite hydrogels in treating bacterial biofilm infections in wounds *in vivo*, we selected male SD rats with three wounds on their backs as a model to evaluate the *in vivo* antibacterial biofilm activity of this system. As shown in Fig. 6a, the male rats were divided into three groups: ① PBS, ② PDA–Cu–PgelMA-T/PgelMA-D, and ③ PgelMA-T/PDA–Cu–PgelMA-D. Based on the gradient nanocomposite hydrogels, local continuous application of the dressing effectively inhibited wound infections and promoted healing. Compared to the PBS group with an infected wound area of approximately 15.66 mm^2^ on the 12th day, the infected wound area in the gradient nanocomposite hydrogel PgelMA-T/PDA–Cu–PgelMA-D group was less than 3 mm^2^, indicating that the PgelMA-T/PDA–Cu–PgelMA-D group could effectively disrupt bacterial biofilms and accelerate the healing of infected wounds (Fig. 6b). By collecting wound tissue treated with gradient hydrogel, we quantified the bacteria at different infection sites among the groups. The bacterial colony count of *S. aureus* in the aforementioned treated groups was only 9.63% of that in the PBS group within the rat's body (Fig. 6c and S4†). Additionally, we assessed the detailed pathological characteristics of the wound sites treated with gradient hydrogel through hematoxylin and eosin staining (H&E staining). In the wounds treated with gradient hydrogel, the epidermal layer remained intact, inflammation cells decreased, and evident granulation tissue was observed, as depicted in Fig. 6d and S5.† In contrast, the other groups exhibited fragmented epidermal layers and more inflammation cells after injury. Inflammatory cytokines such as TNF-α, IL-6, and IL-1β play a significant role in the inflammatory response during bacterial biofilm infections. The histological image in Fig. 6d shows that TNF-α and IL-6 levels (Fig. S6†) in rats treated with gradient hydrogel PgelMA-T/PDA–Cu–PgelMA-D were lower than in the other groups. These results indicate that gradient hydrogel treatment can accelerate wound healing in rats with *S. aureus* biofilm infections by reducing the levels of inflammatory cytokines.

## 4. Conclusion

In this study, we designed a gradient nanocomposite hydrogel based on a simple two-component system, consisting of copper-doped polydopamine nanoparticles and GelMA. Under ultraviolet light treatment, we successfully prepared this gradient composite hydrogel. This gradient hydrogel features a porous structure on the upper side and a relatively dense structure on the lower side. This design not only ensures excellent swelling properties but also guarantees effective contact between the functional antibacterial units and bacteria, resulting in good antibacterial performance. Furthermore, this nanocomposite hydrogel ensures biocompatibility and can effectively combat bacterial biofilms, thereby accelerating the healing of *in vivo* infected wounds. We believe that this research provides a valuable approach for designing multifunctional hydrogels and offers new strategies for developing innovative medical dressings.

## Author contributions

Jiawei Zhu: investigation, validation, writing – original draft, writing – review & editing. Anli Wang: writing – review & editing. Xingguo Miao: investigation, methodology. Hui Ye: writing – review & editing. Shuo Pan: investigation, methodology. Chengxi Zhang: writing – review & editing. Qiuping Qian: writing – original draft, funding acquisition, validation, resources, conceptualization, supervision. Feifei Su: validation, resources.

## Conflicts of interest

There are no conflicts to declare.

## Supplementary Material

RA-013-D3RA06034A-s001
