# Supplementary material for: Harnessing gradient gelatin nanocomposite hydrogels: a progressive approach to tackling antibacterial biofilms
Source: RSC Adv. 2023 Oct 17;13(43):30453–61. doi: 10.1039/d3ra06034a (PMC10580021; doi:10.1039/d3ra06034a)
Supplement: RA-013-D3RA06034A-s001 [file RA-013-D3RA06034A-s001.pdf]

Electronic Supplementary Material (ESI) for RSC Advances.
This journal is © The Royal Society of Chemistry 2023

Supporting Information for:

Harnessing gradient gelatin nanocomposite hydrogels: a progressive approach to tackling antibacterial biofilms

Jiawei Zhu,[a] Anli Wang,[a] Xingguo Miao,[a] Hui Ye,[a] Shuo Pan,[d] Chengxi Zhang,[e] Qiuping Qian ,*[a,b,c] and Feifei Su *[a]

[a] Infectious Disease Department, Wenzhou Central Hospital, Wenzhou 325099, Zhejiang, People's Republic of China. Email: feifeisuzs@163.com

[b] Zhejiang Engineering Research Center for Tissue Repair Materials, Wenzhou Institute, University of Chinese Academy of Sciences, Wenzhou 325000, Zhejiang, People's Republic of China. Email: qianqp@ucas.ac.cn

[c] Departamento de Química Física and Biomedical Research Center (CINBIO), Universidade de Vigo, 36310, Vigo, Spain

[d] Wenzhou Medical University, Wenzhou 325000, Zhejiang, People's Republic of China.

[e] School of Materials Science and Engineering, Shandong Jianzhu University, Jinan 250101, China.

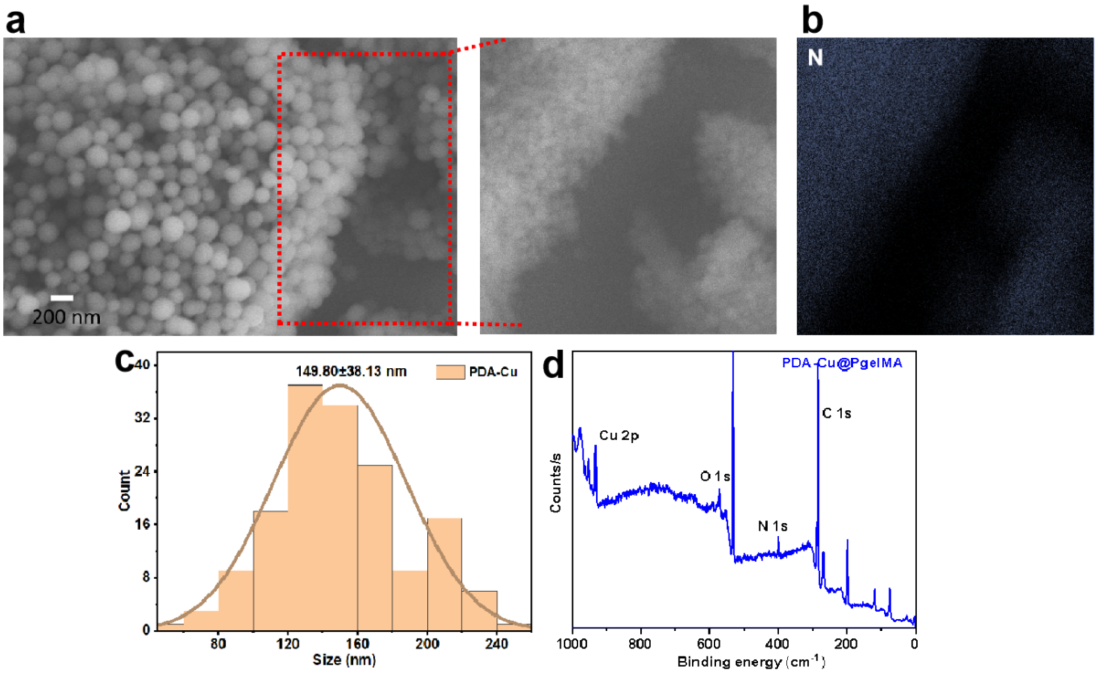

Fig.S1 SEM and element mapping images of PDA-Cu nanoparticles (a and b). Image J analysis of the size of PDA-Cu (c). Full scan XPS survey spectrum and the high-resolution spectra of PDA-Cu@PgelMA (d).

Hydrogel groups	GelMA (5%)	GelMA (10%)	PDA-Cu (1 mg/mL)	PDA-Cu (10 mg/mL)	LAP (20 mg/mL)
1-PgelMA-5%	√				√

2-PgelMA-10%	√			√
3-PDA-Cu@PgelMA-01	√	√		√
3-PDA-Cu@PgelMA-02	√		√	√
4-PGelMA-T(top)/PDA-Cu@PGelMA-D(down)	√		√	√
5-PDA-Cu@PDA-Cu@PGelMA-T/PGelMA-D	√		√	√

Table S1. hydrogel groups.

Sample quality M0 (g)	0.0254
Test solution element coenctration Cu (mg/L)	Cu
Sample elemental content (%)	0.061867

Table S2. ICP-MS analysis of PDA-Cu.

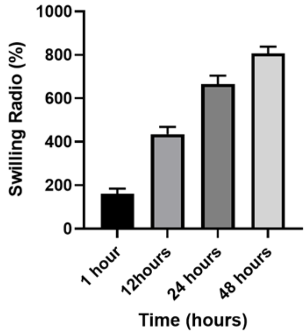

Fig.S2 The hygroscopicity of gradient gelatin nanocomposite hydrogel (n = 3)

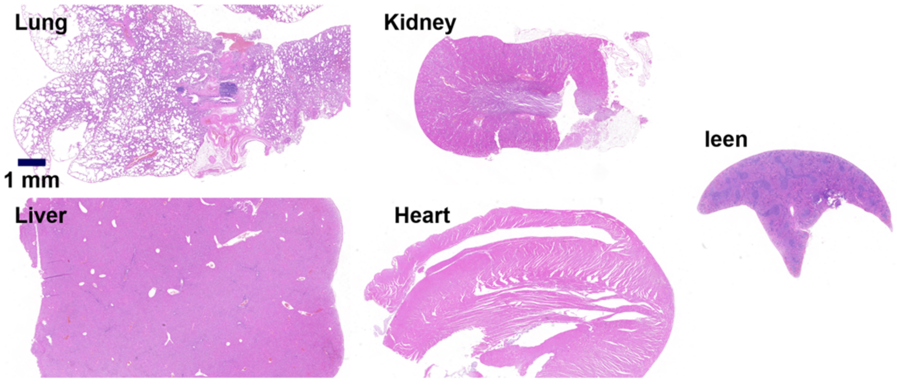

Fig.S3 Microscopy images of hematoxylin and eosin (H&E) stained sections of major organs of rats after 7 days of subcutaneous implantation of gradient gel.

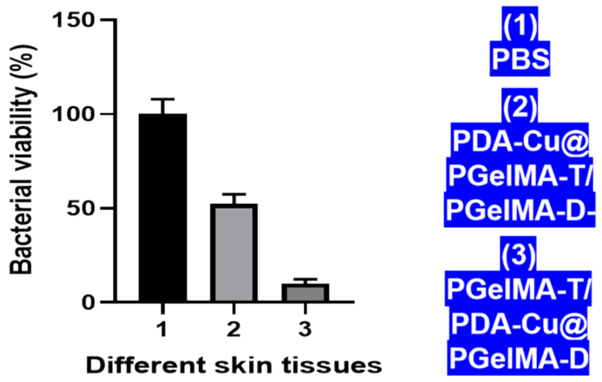

Fig.S4 Evaluation of bacterial colonies inside the infected skin after 6 d treatment (n = 3).

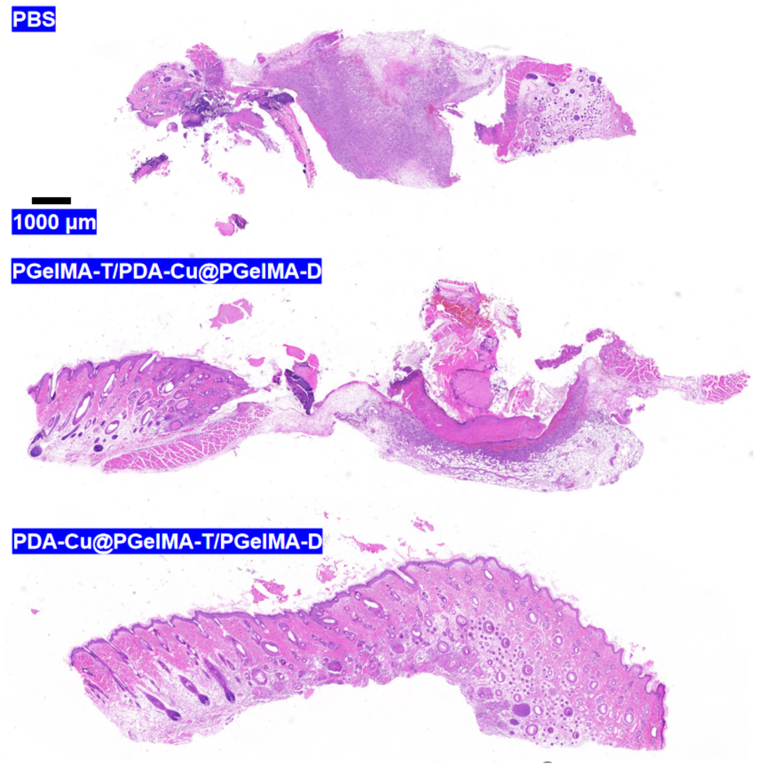

Fig.S5 Microscopy images of hematoxylin and eosin (H&E) stained sections of wound tissues.

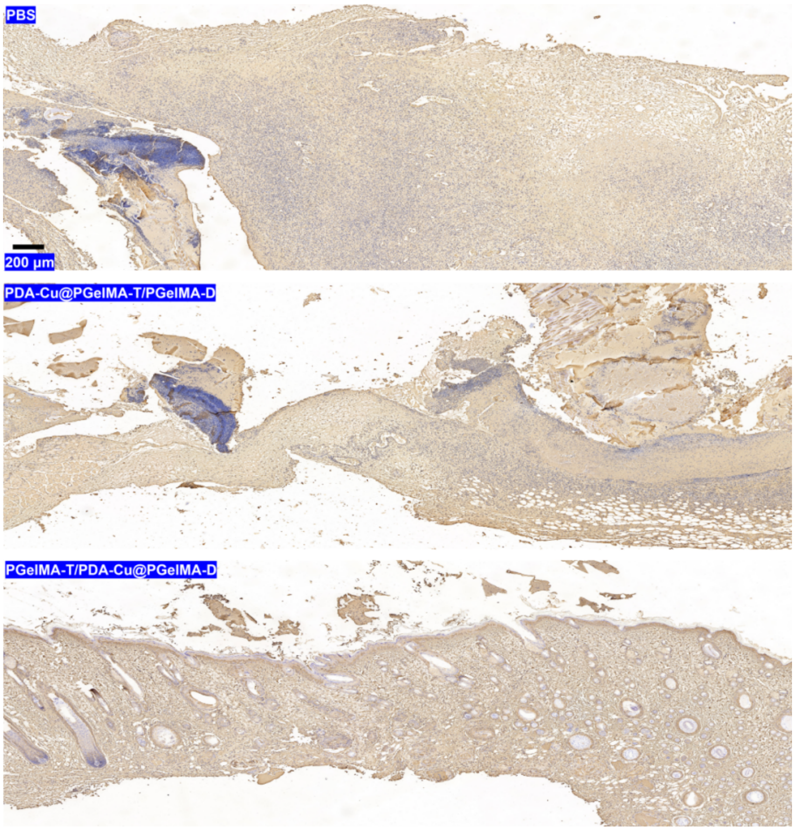

Fig.S6 Infected skin wound tissue evaluated by IHC of IL-6 a after treatment.
